# Supplementary material for: From sequence to enzyme mechanism using multi-label machine learning
Source: BMC Bioinformatics. 2014 May 19;15:150. doi: 10.1186/1471-2105-15-150 (PMC4229970; doi:10.1186/1471-2105-15-150)
Supplement: Additional file 2 — Java code of ml2db. Additional file ml2db_code.tar.gz contains the Java source code to run the multi-label machine learning experiments and save the results to database. The code’s Javadoc is included. [file 1471-2105-15-150-S2.zip › additional file 2/ml2db/ml2db/doc/mulan/evaluation/measure/class-use/Measure.html]

Uses of Interface mulan.evaluation.measure.Measure


JavaScript is disabled on your browser.


- Overview
- Package
- Class
- Use
- Tree
- Deprecated
- Index
- Help

- Prev
- Next

- Frames
- No Frames

- All Classes

## Uses of Interface mulan.evaluation.measure.Measure

- Packages that use Measure

  | Package | Description |
  | --- | --- |
  |  |  |
  | --- | --- |
  | mulan.data |  |
  | mulan.evaluation |  |
  | mulan.evaluation.measure |  |
- - ### Uses of Measure in mulan.data

    Methods in mulan.data that return Measure

    | Modifier and Type | Method and Description |
    | --- | --- |
    |  |  |
    | --- | --- |
    | `Measure` | GreedyLabelClustering.`getMeasure()` |

    Methods in mulan.data with parameters of type Measure

    | Modifier and Type | Method and Description |
    | --- | --- |
    |  |  |
    | --- | --- |
    | `void` | GreedyLabelClustering.`setMeasure(Measure measure)` |
  - ### Uses of Measure in mulan.evaluation

    Methods in mulan.evaluation that return types with arguments of type Measure

    | Modifier and Type | Method and Description |
    | --- | --- |
    |  |  |
    | --- | --- |
    | `java.util.List<Measure>` | Evaluation.`getMeasures()` Returns the evaluation measures |

    Method parameters in mulan.evaluation with type arguments of type Measure

    | Modifier and Type | Method and Description |
    | --- | --- |
    |  |  |
    | --- | --- |
    | `MultipleEvaluation` | Evaluator.`crossValidate(MultiLabelLearner learner, MultiLabelInstances data, java.util.List<Measure> measures, int someFolds)` Evaluates a `MultiLabelLearner` via cross-validation on given data set using given evaluation measures with defined number of folds and seed. |
    | `Evaluation` | Evaluator.`evaluate(MultiLabelLearner learner, MultiLabelInstances data, java.util.List<Measure> measures)` Evaluates a `MultiLabelLearner` on given test data set using specified evaluation measures |

    Constructor parameters in mulan.evaluation with type arguments of type Measure

    Constructor and Description

    `Evaluation(java.util.List<Measure> someMeasures,
    MultiLabelInstances data)`

    Creates a new evaluation object by deep copying the measure objects that
    are given as parameters
  - ### Uses of Measure in mulan.evaluation.measure

    Classes in mulan.evaluation.measure that implement Measure

    | Modifier and Type | Class and Description |
    | --- | --- |
    |  |  |
    | --- | --- |
    | `class` | `AveragePrecision` Implementation of the average precision measure. |
    | `class` | `BipartitionMeasureBase` |
    | `class` | `ConfidenceMeasureBase` |
    | `class` | `Coverage` Implementation of the coverage measure. |
    | `class` | `ErrorSetSize` Measure based on the ErrorSetSize ranking loss function |
    | `class` | `ExampleBasedAccuracy` Implementation of the example-based accuracy measure. |
    | `class` | `ExampleBasedBipartitionMeasureBase` Base class for example-based bipartition measures |
    | `class` | `ExampleBasedFMeasure` Implementation of the example-based F measure. |
    | `class` | `ExampleBasedPrecision` Implementation of the example-based precision measure. |
    | `class` | `ExampleBasedRecall` Implementation of the example-based recall measure. |
    | `class` | `ExampleBasedSpecificity` Implementation of the example-based recall measure. |
    | `class` | `GeometricMeanAverageInterpolatedPrecision` Implementation of GMAiP (Geometric Mean Average Interpolated Precision) |
    | `class` | `GeometricMeanAveragePrecision` Implementation of GMAP (Geometric Mean Average Precision) |
    | `class` | `HammingLoss` Implementation of the Hamming loss function. |
    | `class` | `HierarchicalLoss` Implementation of the Hierarchical loss measure. |
    | `class` | `IsError` Measure based on the IsError ranking loss function |
    | `class` | `LabelBasedAUC` Implementation of the label-based macro precision measure. |
    | `class` | `LabelBasedAveragePrecision` |
    | `class` | `LabelBasedBipartitionMeasureBase` Base class for label-based bipartition measures |
    | `class` | `LabelBasedFMeasure` Base implementation of the label-based macro/micro f-measures. |
    | `class` | `LabelBasedPrecision` Base implementation of the label-based macro/micro precision measures. |
    | `class` | `LabelBasedRecall` Common class for the micro/macro label-based recall measures. |
    | `class` | `LabelBasedSpecificity` Common class for the micro/macro label-based recall measures. |
    | `class` | `LossBasedBipartitionMeasureBase` |
    | `class` | `LossBasedRankingMeasureBase` |
    | `class` | `MacroAUC` Implementation of the macro-averaged AUC measure. |
    | `class` | `MacroFMeasure` Implementation of the macro-averaged f measure. |
    | `class` | `MacroPrecision` Implementation of the macro-averaged precision measure. |
    | `class` | `MacroRecall` Implementation of the macro-averaged recall measure. |
    | `class` | `MacroSpecificity` Implementation of the macro-averaged recall measure. |
    | `class` | `MeanAverageInterpolatedPrecision` Implementation of MAiP (Mean Average Interpolated Precision) |
    | `class` | `MeanAveragePrecision` Implementation of MAP (Mean Average Precision) |
    | `class` | `MeasureBase` |
    | `class` | `MicroAUC` Implementation of the micro-averaged AUC measure. |
    | `class` | `MicroFMeasure` Implementation of the micro-averaged precision measure. |
    | `class` | `MicroPrecision` Implementation of the micro-averaged precision measure. |
    | `class` | `MicroRecall` Implementation of the micro-averaged recall measure. |
    | `class` | `MicroSpecificity` Implementation of the micro-averaged recall measure. |
    | `class` | `OneError` Measure based on the one error loss function |
    | `class` | `RankingLoss` Implementation of the ranking loss measure. |
    | `class` | `RankingMeasureBase` |
    | `class` | `SubsetAccuracy` Implementation of the subset accuracy measure. |

    Methods in mulan.evaluation.measure that return Measure

    | Modifier and Type | Method and Description |
    | --- | --- |
    |  |  |
    | --- | --- |
    | `Measure` | Measure.`makeCopy()` Creates a deep copy of the given measure using serialization. |
    | `Measure` | MeasureBase.`makeCopy()` |

- Overview
- Package
- Class
- Use
- Tree
- Deprecated
- Index
- Help

- Prev
- Next

- Frames
- No Frames

- All Classes
